# Regular Consumption of Green Coffee Phenol, Oat β-Glucan and Green Coffee Phenol/Oat β-Glucan Supplements Does Not Change Body Composition in Subjects with Overweight and Obesity

**DOI:** 10.3390/foods11050679

**Published:** 2022-02-25

**Authors:** Joaquín García-Cordero, José Luis Sierra-Cinos, Miguel A. Seguido, Susana González-Rámila, Raquel Mateos, Laura Bravo-Clemente, Beatriz Sarriá

**Affiliations:** 1 Department of Metabolism and Nutrition, Institute of Food Science, Technology and Nutrition (ICTAN-CSIC), Spanish National Research Council (CSIC), José Antonio Nováis 10, 28040 Madrid, Spain; j.garcia@ictan.csic.es (J.G.-C.); m.seguido@ictan.csic.es (M.A.S.); s.gonzalez@ictan.csic.es (S.G.-R.); raquel.mateos@ictan.csic.es (R.M.); lbravo@ictan.csic.es (L.B.-C.); 2 Department of Nutrition and Food Science I, School of Pharmacy, Complutense University of Madrid (UCM), Ciudad Universitaria, s/n, 28040 Madrid, Spain; 3 Department of Health Science, School of Health Science, Isabel I International University of Burgos (Ui1), 76 Fernán González St., 09003 Burgos, Spain

**Keywords:** green coffee, beta-glucan, food supplement, polyphenols, overweight, BMI, skinfolds, physical activity

## Abstract

Many in vitro and in vivo studies support that green coffee polyphenols (GCP) and beta-glucans (BG) present important hypolipidaemic and hypoglycaemic effects. However, their weight-management/-reducing properties are less clear. Considering that these compounds act on different metabolic pathways, their combination could increase their beneficial health effects. The aim of the present study was to investigate the effects of regularly consuming supplements containing GCP, BG or the novel GCP/BG combination on body composition in overweight/obese subjects without changing their dietary and physical activity habits, hence addressing the difficulty to adapt to lifestyle changes. A randomised, cross-over, blind trial was carried out in 29 volunteers who consumed GCP (300 mg), BG (2.5 g) or GCP/BG (300 mg + 2.5 g) twice a day for 8 weeks. At the beginning and end of each of the interventions, body weight, body mass index, body fat%, intracellular and extracellular water, skinfolds (tricipital, bicipital, subscapularis, suprailiac, leg and thigh) and body circumferences (waist, hip, thigh, calf, branchial) were measured. Along the study, volunteers filled out 72 h dietary records, and physical activity was measured using accelerometers. The results show that dietary intake and physical activity were unchanged throughout the study; however, there were no changes in any of the body composition parameters analysed with any of the food supplements. In conclusion, the regular intake of GCP, BG and GCP/BG, without changes in diet and physical activity, is not an efficient strategy to lose weight or induce other positive changes in body composition, although results should be taken with caution as the study was underpowered.

## 1. Introduction

Over the last 30 years, the prevalence of obesity has increased significantly, and nowadays around 39% of the world’s population is affected. Obesity is more than an imbalance between energy intake and energy expenditure; thanks to the continuous scientific advances in the field, it is known that obesity is a multifactorial disease involving genetic, epigenetic, environmental and socio-economic factors [1]. Moreover, obesity is associated with diverse comorbidities, the most important being coronary disease, cerebrovascular accidents, hypertension, dyslipidemia, type 2 diabetes mellitus, non-alcoholic fatty liver disease, systemic inflammation, disorders of the locomotive system such as osteoarthritis, and even some types of neoplasms (endometrium, breast, liver). These facts have made obesity the second cause of preventable mortality after smoking [1,2]. In view of the alarming increase in the number of cases and the long-term health and socio-economic, among others, repercussions of obesity and obesity-associated non-communicable diseases, new strategies are required, particularly for people with overweight and obesity who present difficulties in adapting to dietary changes and increasing physical activity. In this context, food supplements are challenging tools thanks to their high content in bioactive compounds with beneficial health effects. In fact, bioactive phytochemicals in food supplements have been pointed out as a promising approach to improve patients’ adherence to reducing food and caloric intake [3]. Nowadays, there are numerous supplements for weight loss based on dietary fibre (DF) or polyphenol (PP) rich plant extracts. Besides enhancing metabolic rate, these naturally derived food supplements may also reduce energy intake [4] or induce satiety [3].

Regarding DF, both soluble (SDF) and insoluble (IDF) dietary fibre may help to lose weight, interfering in the digestion and absorption of macronutrients, delaying gastric emptying and increasing satiety. However, only SDF can be metabolised by the intestinal microbiota and induce prebiotic effects by promoting the growth of lactic acid bacteria and bifidobacteria [5,6]. The gut microbiota has been studied in the last years as a factor involved in the onset of overweight. One of the first studies that approached the relationship between gut microbiota and weight gain showed an increased capacity to harvest energy from the diet in obese ob/ob mice [7]. The mechanism that explains the gut microbiota’s influence on energy harvesting is not well known, but it seems to be related with the capacity to metabolise short-chain fatty acids (SCFA) from a wide variety of dietary polysaccharides, meaning that SCFA may serve as the main source of energy for the colonocytes and as metabolic modulators via indirect or direct activation of the AMP-activated protein kinase (AMPK) in the liver [8,9]. In a more recent study carried out in twin pair mice discordant for obesity, the intervention with a low-fat, high-fibre diet prevented the colonization of the obese-microbiota into the lean mice as efficiently as a high-fat, low-fibre diet [10].

Among the SDF, beta-glucan (BG) stands out because of the capacity to form viscous solutions at low concentrations and high fermentation capacity in the colon [11]. BG has been associated with numerous positive health effects, which have been recently reviewed in García-Cordero et al. [12], outstanding its hypolipidaemic [11,13] and hypoglycaemic properties [11,14,15]. Given this strong evidence, the European Food Safety Authority (EFSA) has issued favourable opinions regarding the health effects of BG, stating that regular consumption of at least 3 g/d of BG contributes to the maintenance of normal blood cholesterol levels [16,17]. In addition, considering BG’s hypoglycaemic effects, another favourable opinion was issued establishing the efficiency of the consumption of 4 g of BG for every 30 g of digestible carbohydrates for reducing post-prandial glycaemic responses [18]. However, a cause-and-effect relationship between BG consumption and achievement of a normal body weight has not been established [16], causing controversy surrounding the effects of BG on anthropometric parameters. According to a recent meta-analysis of randomised controlled trials on the effects of cereal BG consumption on body weight, body mass index (BMI), waist circumference (WC) and total energy intake, BG seems to reduce BMI and body weight, but not WC and caloric intake [19]. In contrast, in mildly hypercholesterolemic individuals who consumed 3 g/d of a high molecular weight BG for 5 weeks, positive shifts in BMI and WC were reported, which were correlated with favourable changes in microbiota composition (increase in Bacteroides) [20]. Similarly, a significant improvement in weight, BMI, visceral fat and WC was described in patients with WC ≥ 85 cm for men or ≥90 cm for women and BMI ≥ 24 kg/m^2^ following the intake of 4.4 g/day of BG-rich barley for 12 weeks [21]. Accordingly, in a study carried out in overweight men who either consumed an American Heart Association Step II diet including whole wheat bread, or the Step II diet containing high levels of monounsaturated fatty acids plus bread containing 6 g of BG (Nutrim-OB)/d for 8 weeks, a significant decrease in body weight and BMI was observed in both groups, but it was more prominent in those who consumed bread with BG [22]. To end, in another study, the consumption of 3 g/d of a polysaccharide-rich food supplement, LipiGo^®^, comprising a specific β-glucan–chitin–chitosan fraction obtained from the chemical hydrolysis of *Saccharomyces cerevisiae*, which contained 22.8 g BG/100 g of dry LipiGo^®^, for 12 weeks, resulted in a slight reduction in body weight, WC and body fat compared to baseline [23].

Among the food supplements based on PP-rich plant extracts, outstand those based on green coffee extracts obtained from unroasted coffee beans, particularly rich in hydroxycinamates. Hydroxycinnamates present a wide range of beneficial health properties, revised in García-Cordero et al. [12], including obesity prevention and weight control. These antiobesity properties may be associated with the capacity of these phenols to regulate satiety via increased sensitivity to leptin [24] and glucagon-like peptide-1 (GLP-1) [25], delaying gastric emptying [26] and also with influence on the intestinal microbiota by increasing metabolic activity and the count of *Bifidobacterium* spp. [27]. Hydroxycinnamates’ weight-lowering properties have also been described in studies with obese mice, where the administration of foods rich in hydroxycinnamates such as wine, green coffee and yerba mate induced a decrease in body weight and the accumulation of visceral and hepatic fat (steatosis) as well as lowered the degree of inflammation [28]. In another study, the inhibition of adipocyte differentiation and body weight gain was described after the administration of an aqueous extract of hulled barley via intraperitoneal injections of 15 or 50 mg/kg to high-fat-diet-induced obese mice [29]. In human studies carried out in overweight volunteers, after the consumption of 400 mg/day of Moro orange juice (*Citrus sinensis* (L.) Osbeck), particularly rich in anthocyanins, hydroxycinnamic acids and flavone glycosides, a significant reduction in BMI, WC and hip circumference after 4-week and 12-week treatments, in comparison with the placebo group, was described [30]. Accordingly, a reduction in body weight and fat was observed after drinking 11 g/d of hydroxycinnamic-acid-enriched instant coffee versus normal instant coffee for 12 weeks [31]. Moreover, in a randomised, double-blind, placebo-controlled clinical trial carried out in patients with impaired glucose tolerance, the group that consumed 400 mg of chlorogenic acid three times per day for 12 weeks showed a significant decrease in body weight, BMI, WC, as well as other cardiometabolic parameters compared to the placebo group [32]. Finally, in normoweight healthy and hypercholesterolaemic subjects, the consumption of a hydroxycinnamate-rich green/roasted (35/65) coffee blend for 8 weeks resulted in decreased body weight, BMI and body fat percentage [33,34], pointing to potential beneficial effects of GCP in obesity.

These scientific pieces of evidence support the hypothesis of the present study, that food supplements containing BG and hydroxycinnamates could be used as a strategy to combat overweight and obesity, and that the combination of BG and hydroxycinnamates could have synergic effects, thus being a more efficient nutritional tool to fight these pathologies and their comorbidities. Therefore, the aim of the present study was to investigate the effects of the regular consumption of food supplements containing oat BG, hydroxycinnamates from a GCP extract or the combination of both on body composition in subjects with overweight or obesity. Participants were asked not to change their dietary and physical activity habits so that the observed modifications could be attributed to the food supplements in order to assess if this strategy would be efficient for body weight control without lifestyle changes.

## 2. Materials and Methods

### 2.1. Subjects

Our study population consisted of 29 overweight/obese volunteers (17 male/12 female) with a mean age of 45.2 ± 1.8 (range 28–59 years) and a mean body mass index (BMI) of 30.1 ± 0.6 kg/m^2^. The recruitment was performed mainly by placing advertisements at the Institute of Food Science, Technology and Nutrition (ICTAN-CSIC), at Complutense University of Madrid (UCM) campus and health centres in Madrid, also using social networks to improve the distribution of the advertisement. Although the study of the effects of gender was not an objective for this study, we aimed at gender parity during recruitment. Inclusion criteria were as follows: men and women between 18 and 60 years old and with a BMI 25–35 kg/m^2^. Exclusion criteria were as follows: suffering any chronic pathology other than overweight, obesity or pre-diabetes, smoking, vegetarianism, pregnancy/lactation in women, and taking antibiotics, dietary supplements or hormones.

The study was approved by the Clinical Research Ethics Committee of Hospital Universitario Puerta de Hierro, Majadahonda in Madrid (Spain) and the Bioethics Committee of Consejo Superior de Investigaciones Científicas (CSIC). It also followed the guidelines laid down in the Declaration of Helsinki for experiments in humans. Prior to the start of the study, signed informed consent was obtained from all volunteers. The study was registered in ClincalTrials (NCT05009615).

### 2.2. Food Supplements

To carry out the study, an oat BG concentrate (B-Can^TM^) was used, provided by Garuda International, Inc. (Exeter, CA, USA: data on molecular weight and density are according to suppliers’ information). The product had a 70% BG concentration, a molecular weight between 100 and 200 kg/mol and a density of 0.4–0.5 g/mL. Considering the richness of 70% in B-Can™, 3.57 g were added to the formulation to ensure that each sachet of the supplement provided 2.5 g BG. The physicochemical properties (swelling, water-holding capacity, and oil-holding capacity), viscosity, and the total content in SDF and IDF were measured in our facilities at ICTAN and are described in Redondo-Puente et al. [35] and Mateos et al. [36]. The decaffeinated green coffee extract employed in the preparation of the food supplements was provided by Quimifarma Laboratorios S.L. (Toledo, Spain), and its phenolic concentration was determined in our group [36], so that, considering the richness of 45.8% of PP analysed in the commercial extract, 0.66 g of GCP was added to the formulation of each sachet to provide 300 mg of phenolic compounds. Decaffeinated green coffee was used in order to avoid problems in case of hypertension and because it allowed for using higher quantities of coffee without having to take into account caffeine sensitivity, so the new supplements could be consumed by the largest group of people possible. Food supplements were sealed in individual sachets that contained half of the daily dose and labelled A, B or C for blinding. Volunteers consumed 2 sachets of the corresponding supplement per day, so the daily dose ingested was 5 g/d of BG and 600 mg/d of phenolic compounds. The quantities were selected following the results of a previous dose–response study carried out by our group [36], in which the formulation used in the present study proved to be the most effective in counteracting obesity-related comorbidities.

### 2.3. Study Design

A randomised, cross-over, blind study was conducted over 32 weeks. The randomisation in the study was carried out by a member of the research team who generated random numbers using the Microsoft^®^ Excel 2016 program. In the first intervention, volunteers were randomly assigned to begin with GCP, BG or GCP/BG for 8 weeks; afterwards, all participants continued with a 4-week washout period, followed by a second 8-week intervention with a different food supplement. To end, after a second washout period (4 weeks), the remaining food supplement was consumed for 8 weeks. In each intervention, the volunteers had to consume the corresponding food supplement twice a day, half an hour before breakfast and lunch, by dissolving the product in 250 mL of water. On the two days before each visit, participants had to avoid consuming caffeine and polyphenol-rich foods. Compliance was controlled by calling the volunteers weekly and by counting the number of sachets provided to the volunteers before and after each intervention. After an overnight fast, the participants attended the Human Nutrition Unit (HNU) at the Institute of Food Science, Technology and Nutrition (ICTAN) at the beginning and end of each intervention period, so that anthropometric data and biological samples could be collected.

### 2.4. Dietary Assessment

As aforementioned, during the course of the study, volunteers were asked to keep their dietary habits unchanged to better elucidate the effects of food supplements. To control possible changes, they were asked to fill in a 72 h dietary recall, which included two working days and one weekend day, the days before the visits to the HNU in the three interventions. Energy intake and macronutrient composition were calculated using DIAL software for Windows (version 3.0.0.5; Department of Nutrition, School of Pharmacy (UCM) and Alceingeniería, S.A. Madrid, Spain).

### 2.5. Anthropometric, Physical Activity and Resting Energy Expenditure Analysis

At the beginning of the study, volunteers’ heights were measured using a Holtain precision mechanical stadiometer. At the baseline and the end of each intervention period, volunteers’ body weights were measured using a Tanita scale BC730. BMI was calculated using the formula established by leading scientific institutions (weight (kg)/height (m)^2^). Waist, hip, thigh, calf and brachial perimeters were measured with Cescorf anthropometric tape. Tricipital, bicipital, subscapular, suprailiac, mid-thigh and calf skinfolds were measured using Harpenden skinfold caliper following ISAK protocols (https://www.isak.global/; accessed: 3 December 2018). The sum of the aforementioned skinfolds (SUMM6) was calculated, as well as the sum excluding the thigh and calf measurements (SUMM4). Body fat (%) was also estimated according to the equations by Durnin and Womersley [37] using the biometry data. In addition, total body fat, and intra and extracellular water percentage were also estimated using an INBODY S10 Body Composition Analyser (Microcaya, Bilbao, Spain), which had a multi-frequency and direct segmental bioelectrical impedance.

Physical activity was monitored using ActiGraph wGT3X-BT (CamNtech Ltd. Fenstanton, UK) accelerometers, which captured and recorded high-resolution raw acceleration data that afterwards were converted into a variety of objective activity measures, including energy expenditure and the metabolic equivalent of task (MET) rates. Volunteers were requested to wear the accelerometers 24 h a day during the last week of each intervention period.

To assess resting energy expenditure, indirect calorimetry was analysed using Cosmed Quark RMR (Cosmed, Bicester, UK) with canopy equipment.

### 2.6. Statistical Analysis

In order to estimate the sample size, the G*Power 3.1.9.7 program was used. We considered body weight as the main variable and that the study was a randomised, blind, cross-over intervention. Other premises considered were: a statistical power of 80%, a level of significance of 0.05, a two-tail, a standard deviation of 6.5, and a pre–post difference of 2.5 kg. Considering all these parameters, a final sample size of 38 volunteers was established. The statistical analysis was performed using SPSS software (version 27.0; SPSS, Inc., IBM Company, Armonk, NY, USA).

Before analysing the data, Boxplot analysis was carried out to detect extreme values (outliers) and determine the dispersion and symmetry of the data. In addition, the normality of distribution and homogeneity of variance were evaluated using the Kolmogorov–Smirnov and Levene tests, respectively. A linear mixed model was used to carry out the statistical analysis of the study, as this model allows one to determine the correlated variability and to study the effects of the product consumed, taking into account the order of ingestion. For each variable studied, apart from statistically analysing the baseline values from the start of each intervention, the end values of each intervention, the rates of change were calculated [(end value–baseline value)/baseline value] to better estimate the effects produced by the treatment, evaluating how much the variable increased or decreased with respect to the baseline value. Data are shown in the tables as means ± standard error of the mean.

## 3. Results

### 3.1. Dietary Intake

Volunteers kept their dietary habits unchanged during the 32 week study, according to the 72 h dietary records (Table 1). There were no significant differences at baseline or at the end of each intervention with GCP, BG or GCP/BG related to energy, macro and micronutrient intake. Similarly, there were no statistically significant modifications in the rates of changes in each intervention.

### 3.2. Weight, BMI, Total Body Fat and Intracellular and Extracellular Water

Table 2 shows the main results obtained in relation to body weight, BMI, body fat (calculated with anthropometric formulas and bioimpedance) and water percentage in the volunteers at baseline and at the end of the interventions, together with the corresponding rates of change. There were no significant differences in weight, BMI, water or fat compartments measured by bioimpedance and anthropometric data using the formulas described in Durmin and Womersley [37] at the start and the end of each stage of the study.

### 3.3. Body Skinfold Measurements

Table 3 shows the results obtained in relation to skin folds (tricipital, bicipital, subscapularis, suprailiac, leg and thigh) and the summatory of folds employed (SUMM4 and SUMM6) at the beginning and at the end of the intervention with the three supplements. We did not observe significant changes between these values or in the rates of change after the intake of GC, BG or GC/BG in any of the parameters analysed.

### 3.4. Body Circumference Measurements

Table 4 shows the results of waist, hip, thigh, calf and branchial circumferences, as well as the waist-to-hip ratio, before and after the GC, BG and GC/BG interventions. No significant changes were observed at baseline or at the end of the intervention in any of the different parameters analysed or in the calculated rates of change.

### 3.5. Physical Activity and Resting Energy Expenditure

Table 5 shows the energy expenditure (per day and hour) and metabolic equivalent of task (MET) results at the beginning and at the end of the intervention with each dietary supplement. No significant changes between the different treatments at the baseline, at the end of the intervention, or among the rates of change were obtained.

Indirect calorimetry is based on the principle of gas exchange, and breathing in a calorimeter produces the depletion of O_2_ and the accumulation of CO_2_ in the air camera. In our study, the measurement of O_2_ and CO_2_ was 0.28 ± 0.01 mL/min/m^2^ and 0.23 ± 0.01 mL/min/m^2^, respectively. Considering these results, the average resting energy expenditure was estimated to be 1546 ± 110 and 1964 ± 138 kcal/day, for women and men respectively.

## 4. Discussion

Unquestionably, limiting the energy intake from total fats and sugars, increasing the consumption of fruit and vegetables, as well as legumes, whole grains and nuts, and engaging in regular physical activity are the best strategies to reduce the incidence of overweight and obesity [1]. However, changes in dietary and physical activity patterns are difficult to achieve individually in the long term. Presently, supportive policies at socio-sanitary, environmental, transport, education, and other levels are insufficient to promote healthier lifestyles, especially in urban areas. In this context, alternative strategies such as the consumption of weight-loss food supplements are gaining acceptance. Bearing in mind that overweight and obesity are reaching alarming rates worldwide, as well as the non-communicable diseases associated to obesity, it is urgent to find alternative strategies to combat these pathologies. Therefore, this work was aimed at assessing if consuming supplements with potential weight-management/-lowering properties, such as containing oat BG, GCP or the combination of both, could be an efficient strategy to reduce weight and/or produce other positive changes in body composition in subjects with overweight/obesity without lifestyle changes. If the tested food supplements were effective, they could be particularly interesting for people who have difficulties in changing their dietary or physical activity habits.

### 4.1. Dietary and Physical Activity Habits Analysis

The monitoring of volunteers’ dietary habits showed that the intake of kilocalories, macro- and micronutrients did not change during the study (Table 1). Therefore, we can conclude that volunteers followed the instruction not to change their diet. Moreover, attending to the dietary results, it can be inferred that the food supplements did not induce modifications at this level. The 72 h records show that the average daily caloric intake was 2000–2100 kcal/d. This involves that the average resting metabolic rate of 1814 kcal/d (the mean of 1546 ± 110 and 1964 ± 138 kcal/day for women and men, respectively) represents 85% of the daily caloric intake. Considering that the volunteers’ body weight did not change along the study, this result may be caused by an underestimation of energy intake, and not to the estimation of physical activity and average metabolic rate which were measured with more accurate methods. In relation to the macronutrient intake, our population did not follow the general dietary recommendations established for the adult population, as 40% of the energy of their diet was provided by lipids, instead of the recommended 30–35%, and proteins provided 17.3% of the energy, instead of the 10–15% recommended for the adult Spanish population [38]. Moreover, looking into the dietary lipid composition, the consumption of saturated fatty acids was higher than the recommended at the expense of monounsaturated and polyunsaturated fatty acids [34]. This dietary pattern may be associated with higher consumption of meat and fatty foods, a tendency that has been often observed in recent years in the Spanish population, who is moving away from the Mediterranean diet and approaching the less healthy Western dietary pattern associated with obesity and other metabolic diseases, such as diabetes and hypertension [39].

Regarding physical activity, there were no differences between the interventions, so the volunteers also followed the study instructions in this respect (Table 5). Physical activity energy expenditure and especially the MET ratio of the study population (Table 5) correspond to a sedentary lifestyle, where most of the energy is spent on activities such as writing, desk-work and computer activities [40]. In previous nutritional studies carried out by our research group, physical activity had been estimated using an adapted version of the Minnesota Leisure Time Physical Activity Questionnaire by Martínez-González et al. [41] or by questionnaires designed by the research team to be completed on three representative days (two working and one weekend days), so that the questionnaire covered both occupational and sport-related activities [42]. Considering the limitations observed when these questionnaires were used to estimate physical activity due to incomplete or unreal answering, as volunteers tended to overestimate their physical activity, in the present study, accelerometers were used, which provided accurate measures of physical activity and sleep/wake assessment for 24 h, including primary acceleration, energy expenditure, MET ratios, steps taken and intensity of physical activity. Certainly, in the present study, the physical activity measurement was more accurate than the estimation made using questionnaires; however, interindividual variability was particularly high. Volunteers indicated that it was uncomfortable to permanently wear the accelerometers; therefore, this method requires higher compliance from the volunteers and may be considered more invasive, as participants even described the feeling of being “observed”. On the other hand, it is well known that resting energy expenditure changes from one person to another due to physiological characteristics such as body size, corporal composition, age, sex and production of hormones. These factors could explain the high variability also observed regarding resting energy expenditure in the present study. However, due to the relatively low number of volunteers, we did not separate the participants according to sex or any of the factors mentioned above. Considering the dietary and physical activity results together, volunteers followed a pro-obesogenic lifestyle, and there were no changes in dietary or physical activity that could affect body composition during the study.

### 4.2. Effects of Food Supplements BG and GC on Body Composition

Sound scientific evidence supports that BG may promote health by improving glucose and lipid metabolism as evidenced by the positive opinions issued by EFSA [16,17,18]. However, in 2006, EFSA did not support the effects on BG and the achievement of normal weight [16]. Since then, more clinical trials have been carried out on this subject, some of which have shown weight-, BMI- as well as WC reducing effects. Among these studies, outstands the intervention carried out in hypercholesterolaemic subjects who consumed 3 g/d of high molecular weight barley BG for 5 weeks and described a reduction in BMI and WC that was correlated with an improvement in the microbiota profile [20]. In a study carried out in volunteers with overweight and obesity, after consumption for 12 weeks of 3 g/d of a BG supplement obtained from the chemical hydrolysis of *Saccharomyces cerevisiae*, a statistically significant reduction in BMI, body weight (−1.8 kg) and WC was described [23], but it is important to mention that this reduction was not clinically significant, meaning that the results of the study did not translate into significant improvements in the health status. In turn, in another study in overweight/obese subjects, a statistically and clinically significant reduction in body weight (−2.6 kg), compared to the placebo, was reported after an intervention with 3 g/d of oat cereal rich in BG for 12 weeks without changes in WC [43]. In contrast, some studies do not support BG’s weight-lowering effects, such as that carried out in overweight women who showed a significant reduction in weight and WC after treatment for 3 months with two energy-deficient diets including 5–6 g and 8–9 g of BG/day, respectively, but this reduction was also observed in the control group (who consumed the same type of energy-deficient diet without BG). These authors concluded that the BG supplementation did not improve the effectiveness of the hypocaloric diet in reducing weight and other anthropometric parameters [44]. In accordance with this, in a recent meta-analysis of randomised clinical trials with dietary supplements containing isolated compounds on the effectiveness of weight loss [45], two out of three clinical trials using BG reported a statistically, but not clinically, significant reduction in weight after a treatment with 4.4 g/d of barley enriched with BG for 12 weeks [21] or with a supplement product containing 22.8 g BG/100 g of dry product (3 g of supplement per day) during 12 weeks in 56 volunteers [23]. The results of the present trial are in line with the last group of studies and suggest that BG, at the dose of 5 g/d for 8 weeks, does not induce changes in the end values or rates of change of body weight, BMI, fat% or the skinfolds or body circumferences measured at the end of the interventions. Bearing in mind the aforementioned studies, the lack of effects could be in part due to the length of intervention (under 12 weeks as in [23]) or to the type of BG product used, although the amount and concentration of oat BG were carefully selected, attending to the results of a dose–response study carried out by our group. In this parallel, randomised, blind study [36], two doses (3 g/d or 5 g/d) of two types of oat BG (35% and 70% BG) were used together with a fixed amount of 600 mg of decaffeinated green coffee hydroxycinnamates. Considering that the study followed a non-experimental pre-test/post-test design, in which the control was each individual at baseline, an improvement in total body fat percentage, visceral fat percentage, and waist and hip circumferences was observed after six weeks with the four treatments without differences among the food supplements. Afterwards, when the rates of change were calculated as [(end value-baseline value)/baseline value] and Bonferroni correction paired *t*-test was carried out, only the change rate of total body fat% was significantly lower with 5 g/d of the 70% BG [36], and thus that was the BG dose and product selected for the present study.

Similar disagreement exists regarding the antiobesity effects of hydroxycinnamates. In the recent systematic review carried out by Coman and Vodnar [46] on the effects of hydroxycinnamic acids on human health, only two studies showed a significant decrease in weight, BMI and body fat. One was carried out in overweight volunteers treated with 10 g/d of instant coffee enriched with chlorogenic acid (90–100 mg of chlorogenic acid per 2200 mg of coffee) for 12 weeks, versus normal instant coffee. The average weight loss was 5.4 and 1.7 kg with the chlorogenic acid rich coffee and with the control drink, respectively [31]. The other intervention was carried out in patients with impaired glucose tolerance who consumed 400 g of chlorogenic acid three times per day for 12 weeks; after the treatment, a reduction in BMI, body weight and WC compared to the placebo was observed [32]. Contrastingly, there are studies that do not support the antiobesity effects of hydroxycinnamates, such as that carried out by Soga et al. [47] in healthy male volunteers who consumed 329 mg/d of chlorogenic acid for 4 weeks. No significant reduction in body weight, BMI and body fat% compared to the placebo was observed [47]. Accordingly, Bumrungpert et al. [48] did not report a significant reduction in weight, BMI and body fat after a 6-week intervention with 1000 mg per day of ferulic acid presented in oral capsules in hyperlipidemic subjects [48]. This is in line with the results of the present intervention with GCP, providing 600 mg of hydroxycinnamic acids per day, since this food supplement did not produce changes in body weight, BMI, total fat percentage or the skinfolds measured.

### 4.3. Effects of the Food Supplement BG/GC on Body Composition

The idea of combining BG and GC resulted from the attempt to understand if these compounds could exert complementary or synergistic actions, considering that the mechanisms of action of PP and SDF are different, as they affect the absorption and metabolism of nutrients (mainly fats and sugars) differently, with thermogenic effects, promoting satiety, acting as laxatives, etc. (revised in [12]). In a recent study [35], it was described that the regular consumption of GCP/BG could reduce satiety in the short term, which was related with increased leptin levels, as well as appetite in the long term (after 8 weeks), which was associated with lower ghrelin levels. In contrast, these appetite/satiety effects were not observed in the intervention with GCP in the same group of volunteers during the same extension of time (8 weeks). Another reason for studying GCP/BG was that, as far as we know, there are no food supplements that combine GCP and SDF. Should these bioactive compounds have produced synergistic beneficial effects, the combination could have also been used to design functional foods to offer more alternatives for people with overweight/obesity. However, the regular consumption of GCP/BG did not produce any changes in volunteers’ body compositions, likely for the same reasons pointed out above with GCP and BG: the intervention was too short and/or the amount of product studied was not high enough. Moreover, the number of volunteers was lower than the minimum established in the power calculations, and the high interindividual variability should also be taken into consideration. Furthermore, part of the aforementioned controversial effects of BG and GC could be attributed to the fact that the biological activity of BG and hydroxycinnamate supplements depends on the source and the extraction and purification methods used to obtain these compounds [49].

Knowing that the GCP/BG food supplement used here had induced changes in total body fat percentage in the dose–response trial previously described [36], in the present study this parameter was measured by means of the biometry data using the equations by Durnin and Womerstly [37] and by bioimpedance. A good agreement was observed between the two methods; however, none of them showed differences after the intake of the food supplements. The bioimpedance equipment used allowed to measure the water compartment, discriminating between intra- and extracellular water. It was important to assess if there were any changes in the water compartments that could translate into weight changes; however, again, there were no differences between the studied treatments (Table 2). It is well known that subcutaneous fat reflects the amount of fat present in the adipose tissue; in fact, 40–60% of body fat is in the subcutaneous region [50]. In addition, skinfold determinations provide information concerning local fat depots and fat distribution in the body [51]. Therefore, tricipital, bicipital, subscapularis, suprailiac, leg and thigh were measured and no differences were observed. Furthermore, skinfold sums were calculated, which provide an index to determine adiposity, and again the absence of differences between GCP, BG and GCP/BG treatments was confirmed.

Another factor that could also affect the bioactivity of BG, GC and BG/GC is their effects on the gut microbiota. As indicated in the introduction, gut microbiota is linked with weight gain via an increase in the ability of the altered microbiota to extract energy from the food, such as in people with obesity. Moreover, an increase in the Firmicutes:Bacteroidetes ratio in overweight/obese subjects has been described in comparison to healthy individuals [10]. BG can regulate the human microbiota by being a source of energy for various species of bacteria, such as Lactobacilli and Bifidobacteria [52]. Other in vivo and in vitro studies also support that BG may promote the growth of Lactobacilli and Bifidobacteria [53,54,55], and, in addition, a recent clinical trial showed an increase in levels of Lactobacilli, Clostridiaceae (*Clostridium orbiscindens* and *Clostridium* spp.), *Roseburia hominis*, and *Ruminococcus* spp. and a decrease in Firmicutes and Fusobacteria after the administration of whole-grain barley pasta containing 3 g/day of barley BG for 2 months [56]. However, to our knowledge, only in the study by Wang et al. (2016) [20] the changes in gut microbiota induced by the ingestion of BG were associated with anthropometric parameters, meaning that the increase in the proliferation of Bacteroidetes, Bacteroides and Prevotella counts correlated with a positive shift in BMI and WC, along with a reduction in Firmicutes and Dorea counts [20]. On the other hand, regarding hydroxycinnamates, both in vivo and in vitro studies demonstrate the efficacy of these compounds in modulating the gut microbiota (reviewed in Leonard et al. [57]). Among the studies in this review outstands that carried out in C57BL/6J female mice that showed an increment in the population of Akkermansia and a decrease in Firmicutes after a 15-day treatment with 1.8 mg/kg of body weight per day of pure caffeic acid [58], and that carried out in ICR mice that showed an increase in Bacteroidaceae and Lactobacillaceae and a decrease in Desulfovibrionaceae, Ruminococcaceae, Lachnospiraceae and Erysipelotrichaceae populations after daily consumption of 150 mg/kg by body weight of pure chlorogenic acid [59]. However, to our knowledge, only the study carried out by Jaquet et al. [27] in healthy adult volunteers has approached the effects of hydroxycinnamates on the gut microbiota. In that study, daily consumption of three cups of coffee for 3 weeks showed an increment in the population of *Bifidobacterium* spp. [27]. Considering the importance of microbiota composition in obesity, this analysis will be included in new studies that are being carried out in our group.

Limitations: This study was carried out on 29 subjects and thus it was underpowered. It is possible that our study was not long enough, since in a previous 12-week-long intervention significant differences have been observed [23,32], although in the mentioned dose–response study with GCP/BG, statistically significant differences were observed after only 6 weeks [36]. Another reason that could explain the lack of differences may be the high interindividual variability observed due to gender, BMI, age, etc. However, due to the low number of volunteers who participated in the study, we could not make subgroups to study the possible influence of these factors on the observed body composition results. An additional restraint was that the dietary analysis was based on 72 h records that were filled prior to each of the visits to the Human Nutrition Unit, and no food preference or food-frequency questionnaires were considered. Perhaps there were background effects of the diet that were not detected. In addition, the dietary records may lead to inaccurate measurements of food intake, such as the underestimation of energy intake observed. Due to time and economic constraints, it was not possible to carry out the analysis of the microbiota in the study population.

## 5. Conclusions

The regular intake of the studied food supplements containing GCP, BG and GCP/BG, without changes in dietary and physical activity habits, is not an efficient strategy to lose weight or produce other positive changes in body composition in overweight or obese people. However, we do not dismiss the possibility that, if the study had been carried out in a larger group of volunteers, and/or if the interventions had been longer, positive effects would have been observed, considering the health properties of BG and GCP.

## Figures and Tables

**Table 1:** Energy, macronutrient and micronutrient intakes recorded in 72 h dietary records filled in by volunteers during each intervention stage.

	GCP	BG	GCP/BG	*p*
**Energy (Kcal)**				
Baseline	2019 ± 100	2058 ± 109	2023 ± 112	0.971
End	2115 ± 118	1937 ± 80	2128 ± 115	0.537
Rate of change	0.08 ± 0.06	−0.02 ± 0.04	0.08 ± 0.06	0.265
**Protein (g)**				
Baseline	82.8 ± 4.1	84.8 ± 4.7	79.7 ± 3.6	0.759
End	82.6 ± 4.7	85.2 ± 4.1	86.9 ± 4.5	0.796
Rate of change	0.05 ± 0.07	0.04 ± 0.05	0.12 ± 0.06	0.570
**CHO (g)**				
Baseline	199 ± 11	211 ± 13	205 ± 14	0.850
End	204 ± 11	196 ± 12	200 ± 13	0.865
Rate of change	0.09 ± 0.07	−0.01 ± 0.07	0.06 ± 0.09	0.604
**Dietary Fibre (g)**				
Baseline	23.9 ± 1.9	23.6 ± 1.8	22.5 ± 1.6	0.813
End	22.4 ± 1.9	20.6 ± 1.4	21.9 ± 1.4	0.602
Rate of change	0.20 ± 0.21	−0.03 ± 0.08	0.06 ± 0.07	0.331
**Fat (g)**				
Baseline	84.7 ± 5.8	81.9 ± 4.9	86.5 ± 6.3	0.997
End	93.6 ± 6.8	79.9 ± 4.3	96.9 ± 7.2	0.283
Rate of change	0.16 ± 0.08	0.07 ± 0.08	0.18 ± 0.08	0.363
**SFA (g)**				
Baseline	28.01 ± 2.2	26.9 ± 1.7	28.5 ± 2.0	0.798
End	30.9 ± 2.8	25.9 ± 1.7	31.5 ± 2.7	0.381
Rate of change	0.18 ± 0.11	0.08 ± 0.09	0.19 ± 0.11	0.648
**MUFA (g)**				
Baseline	34.5 ± 2.4	34.9 ± 2.3	34.7 ± 2.2	0.942
End	41.9 ± 3.2	34.5 ± 2.1	42.8 ± 3.2	0.185
Rate of change	0.31 ± 0.11	0.11 ± 0.09	0.29 ± 0.09	0.106
**PUFA (g)**				
Baseline	12.8 ± 1.1	12.8 ± 1.2	12.3 ± 1.2	0.923
End	11.7 ± 0.9	12.2 ± 0.9	12.4 ± 0.8	0.828
Rate of change	0.02 ± 0.09	0.09 ± 0.08	0.18 ± 0.11	0.304
**PUFA/SFA**				
Baseline	0.5 ± 0.04	0.4 ± 0.04	0.45 ± 0.04	0.800
End	0.42 ± 0.03	0.5 ± 0.03	0.46 ± 0.04	0.248
Rate of change	0.04 ± 0.09	0.15 ± 0.07	0.22 ± 0.17	0.845
**[PUFA + MUFA]/SFA**				
Baseline	1.8 ± 0.1	1.8 ± 0.1	1.8 ± 0.1	0.995
End	1.9 ± 0.1	1.9 ± 0.1	1.9 ± 0.1	0.998
Rate of change	0.14 ± 0.08	0.09 ± 0.05	0.15 ± 0.08	0.845
**Cholesterol (mg)**				
Baseline	299 ± 26	330 ± 29	329 ± 24	0.728
End	287 ± 29	325 ± 28	366 ± 32	0.205
Rate of change	0.09 ± 0.12	0.1 ± 0.1	0.2 ± 0.2	0.748
**Calcium (mg)**				
Baseline	758 ± 46	775 ± 50	688 ± 45	0.241
End	752 ± 70	727 ± 54	814 ± 69	0.376
Rate of change	−0.01 ± 0.06	−0.05 ± 0.06	0.25 ± 0.13	0.231
**Iron (mg)**				
Baseline	14.2 ± 0.8	14.9 ± 0.9	13.3 ± 0.7	0.336
End	14.4 ± 0.8	12.9 ± 0.6	15.3 ± 1.0	0.049
Rate of change	0.13 ± 0.10	−0.05 ± 0.07	0.20 ± 0.08	0.095

Values represent mean ± standard error of the mean (SEM), *n* = 29. *p* values correspond to the comparison of baseline values, end values or the comparison of the rates of change of GCP, BG and GCP/BG using a linear mixed model analysis. Rate of change represents [(end value − baseline value)/baseline value]. Carbohydrate (CHO); saturated fatty acids (SFA); monounsaturated fatty acids (MUFA); polyunsaturated fatty acids (PUFA).

**Table 2:** Body weight, body mass index (BMI), total body fat, determined by anthropometry or bioimpedance (Fat-BIA), and water percentages at baseline and at the end of the interventions with the food supplements containing green coffee polyphenols (GCP), beta-glucan (BG) and the mixture of GCP/BG.

	GCP	BG	GCP/BG	*p*
**Body Weight (Kg)**				
Baseline	86.4 ± 1.8	86.8 ± 2.3	87.2 ± 4.3	0.976
End	86.3 ± 1.8	86.5 ± 2.4	87.7 ± 4.3	0.936
Rate of change	−0.002 ± 0.002	−0.003 ± 0.003	0.006 ± 0.004	0.177
**BMI (Kg/m^2^)**				
Baseline	30.05 ± 0.48	30.09 ± 0.64	29.9 ± 1.1	0.980
End	30.1 ± 0.5	29.9 ± 0.6	31.1 ± 1.1	0.997
Rate of change	−0.002 ± 0.002	−0.003 ± 0.003	0.006 ± 0.004	0.177
**Fat (%)**				
Baseline	35.2 ± 0.9	35.4 ± 1.2	35.1 ± 1.6	0.988
End	35.2 ± 0.9	35.3 ± 1.2	35.2 ± 1.6	0.999
Rate of change	0.001 ± 0.004	−0.002 ± 0.003	0.005 ± 0.005	0.248
**Fat BIA (%)**				
Baseline	29.01 ± 1.18	29.4 ± 1.5	28.9 ± 2.7	0.996
End	28.7 ± 1.1	29.6 ± 1.5	29.9 ± 2.8	0,833
Rate of change	0.003 ± 0.023	0.08 ± 0.08	0.05 ± 0.03	0.677
**Intracellular water (%)**				
Baseline	26.2 ± 0.8	26.8 ± 0.9	26.7 ± 1.2	0.907
End	26.6 ± 0.8	26.1 ± 0.8	26.4 ± 1.3	0.904
Rate of change	0.02 ± 0.02	−0.02 ± 0.02	−0.01 ± 0.01	0.224
**Extracellular water (%)**				
Baseline	15.7 ± 0.4	16.2 ± 0.5	16.1 ± 0.7	0.918
End	15.8 ± 0.4	15.6 ± 0.5	15.9 ± 0.8	0.925
Rate of change	0.007 ± 0.011	−0.02 ± 0.01	−0.01 ± 0.01	0.312

Values represent mean ± SEM, *n* = 29. *p* values correspond to the comparison of baseline values, end values or the comparison of the rates of change of GCP, BG and GCP/BG interventions using a linear mixed model analysis. Rate of change represents [(end value − baseline value)/baseline value].

**Table 3:** Skinfolds measured at baseline and at the end of each intervention with the food supplements containing green coffee polyphenols (GCP), beta-glucan (BG) and the mixture of GCP/BG.

	GCP	BG	GCP/BG	*p*
**Tricipital (mm)**				
Baseline	22.9 ± 1.1	23.4 ± 1.3	23.3 ± 1.5	0.946
End	22.8 ± 1.04	23.2 ± 1.3	23.3 ± 1.6	0.943
Rate of change	−0.002 ± 0.001	−0.001 ± 0.001	−0.003 ± 0.001	0.311
**Bicipital (mm)**				
Baseline	13.7 ± 0.8	13.1 ± 1.02	12.4 ± 1.3	0.73
End	13.8 ± 0.9	13.3 ± 1.01	12.3 ± 1.3	0.672
Rate of change	0.07 ± 0.02	0.03 ± 0.02	0.01 ± 0.04	0.833
**Subscapularis (mm)**				
Baseline	24.8 ± 1.1	25.4 ± 1.4	24.7 ± 2.3	0.929
End	24.7 ± 1.1	24.9 ± 1.4	25.04 ± 2.33	0.984
Rate of change	−0.06 ± 0.01	−0.02 ± 0.01	0.02 ± 0.02	0.183
**Suprailiac (mm)**				
Baseline	31.1 ± 0.9	31.6 ± 1.2	31.1 ± 2.5	0.943
End	31.5 ± 0.8	31.6 ± 1.2	31.5 ± 2.4	0.998
Rate of change	0.02 ± 0.01	0.004 ± 0.011	0.02 ± 0.02	0.74
**Leg (mm)**				
Baseline	20.9 ± 1.2	20.4 ± 1.4	19.9 ± 1.7	0.902
End	20.7 ± 1.1	21.3 ± 1.5	19.6 ± 1.6	0.716
Rate of change	0.002 ± 0.017	0.05 ± 0.04	−0.01 ± 0.02	0.671
**Thigh (mm)**				
Baseline	29.1 ± 1.3	29.1 ± 1.5	29.9 ± 2.3	0.957
End	29.1 ± 1.3	27.3 ± 1.7	30.1 ± 2.3	0.63
Rate of change	0.001 ± 0.009	−0.04 ± 0.04	0.01 ± 0.03	0.769
**SUMM 4**				
Baseline	92.8 ± 3.2	93.28 ± 4.03	91.5 ± 6.6	0.968
End	92.7 ± 3.2	93.3 ± 4.1	92.2 ± 6.6	0.987
Rate of change	0.001 ± 0.007	0.001 ± 0.007	0.009 ± 0.012	0.149
**SUMM 6**				
Baseline	142.6 ± 5.1	143.3 ± 6.1	141.3 ± 8.9	0.99
End	142.7 ± 5.02	141.6 ± 5.9	141.8 ± 9.01	0.992
Rate of change	0.002 ± 0.006	−0.007 ± 0.009	0.005 ± 0.008	0.579

SUMM 4 = skinfolds sum excluding the mid-thigh and calf measurements, SUMM 6 = total skinfolds sum. Values represent mean ± SEM, *n* = 29. *p* values correspond to the comparison of baseline values, end values or the comparison of the rates of change of GCP, BG and GCP/BG using a linear mixed model analysis. Rate of change represents [(end value − baseline value)/baseline value].

**Table 4:** Circumferences measured at baseline and at the end of the intervention with the food supplements containing green coffee polyphenols (GCP), beta-glucan (BG) and the mixture of GCP/BG.

	GCP	BG	GCP/BG	*p*
**Waist (cm)**				
Baseline	99.9 ± 1.6	100.6 ± 2.2	103.6 ± 4.4	0.615
End	100.1 ± 1.6	100.6 ± 2.1	104.04 ± 4.45	0.584
Rate of change	0.003 ± 0.003	0.001 ± 0.003	0.004 ± 0.004	0.943
**Hip (cm)**				
Baseline	106.5 ± 1.03	106.4 ± 1.2	104.9 ± 1.6	0.715
End	106.4 ± 1.02	106.4 ± 1.2	105.3 ± 1.6	0.837
Rate of change	−0.001 ± 0.001	−0.003 ± 0.002	0.003 ± 0.001	0.105
**Waist-to-hip ratio**				
Baseline	0.94 ± 0.02	0.95 ± 0.02	0.99 ± 0.03	0.418
End	0.94 ± 0.02	0.95 ± 0.02	0.99 ± 0.04	0.443
Rate of change	0.004 ± 0.002	0.001 ± 0.004	0.004 ± 0.003	0.612
**Thigh (cm)**				
Baseline	57.3 ± 0.6	57.8 ± 0.8	56.6 ± 0.9	0.699
End	57.3 ± 0.6	57.2 ± 0.8	56.3 ± 0.8	0.697
Rate of change	−0.005 ± 0.004	−0.009 ± 0.004	−0.005 ± 0.007	0.498
**Calf (cm)**				
Baseline	40.08 ± 0.54	40.05 ± 0.53	39.2 ± 0.7	0.635
End	40.1 ± 0.5	39.9 ± 0.6	39.5 ± 0.7	0.798
Rate of change	0.0007 ± 0.002	−0.004 ± 0.005	0.006 ± 0.003	0.397
**Brachial (cm)**				
Baseline	33.4 ± 0.5	33.5 ± 0.5	33.1 ± 0.9	0.921
End	33.3 ± 0.4	33.4 ± 0.5	33.3 ± 0.9	0.993
Rate of change	−0.0004 ± 0.0027	−0.001 ± 0.004	0.007 ± 0.004	0.213

Values represent mean ± SEM, *n* = 29. *p* values correspond to the comparison of baseline values, end values or the comparison of the rates of change of GCP, BG and GCP/BG using a linear mixed model analysis. Rate of change represents [(end value − baseline value)/baseline value].

**Table 5:** **Physical activity** energy expenditure and metabolic equivalent of task (MET) at baseline and at the end of the interventions with the food supplements containing green coffee polyphenols (GCP), beta-glucan (BG) and the mixture of GCP/BG.

	GCP	BG	GCP/BG	*p*
**Energy Expenditure (Kcal/day)**				
Baseline	392.9 ± 52.2	406.87 ± 51.01	556.01 ± 57.55	0.198
End	453.9 ± 76.8	346 ± 42.7	428.7 ± 65.1	0.513
**Kcal/hour**				
Baseline	17.7 ± 2.4	17.7 ± 2.2	24.0 ± 2.5	0.313
End	19.4 ± 3.4	14.5 ± 1.8	18.1 ± 2.7	0.481
**MET**				
Baseline	1.3 ± 0.1	1.1 ± 0.1	1.1 ± 0.2	0.506
End	1.18 ± 0.05	1.1 ± 0.1	1.1 ± 0.1	0.671

Values represent mean ± SEM, *n* = 29. *p* values correspond to the comparison of baseline values, and end values of GCP, BG and GCP/BG using a linear mixed model analysis. Rate of change represents [(end value − baseline value)/baseline value].

## Data Availability

Data have not been deposited in any repository, yet they can be made available to researchers upon request.
